# Draft genome of tule elk *Cervus canadensis nannodes*

**DOI:** 10.12688/f1000research.12636.2

**Published:** 2017-12-11

**Authors:** Jessica E. Mizzi, Zachary T. Lounsberry, C. Titus Brown, Benjamin N. Sacks

**Affiliations:** 1 Microbiology Graduate Group, University of California, Davis, CA, 95616, USA; 2 Veterinary Genetics Laboratory, University of California, Davis, CA, 95616, USA; 3 Department of Population Health and Reproduction, School of Veterinary Medicine, University of California, Davis, CA, 95616, USA; 4 Department of Population Health and Reproduction and Mammalian Ecology and Conservation Unit, Veterinary Genetics Laboratory, School of Veterinary Medicine, University of California, Davis, CA, 95616, USA

**Keywords:** Cervus elaphus nannodes, genome draft, mammalian genome assembly, tule elk

## Abstract

This paper presents the first draft genome of the tule elk ( *Cervus elaphus nannodes*), a subspecies native to California that underwent an extreme genetic bottleneck in the late 1800s.  The genome was generated from Illumina HiSeq 3000 whole genome sequencing of four individuals, resulting in the assembly of 2.395 billion base pairs (Gbp) over 602,862 contigs over 500 bp and N50 = 6,885 bp. This genome provides a resource to facilitate future genomic research on elk and other cervids.

## Introduction

At the initiation of this project, no genome assembly existed for any member of the deer family (Cerivdae). We therefore sought to generate the first such assembly for the tule elk ( *Cervus canadensis nannodes*). We note that after we completed our project and submitted the intial draft of this manuscript, a full assembly of red deer ( *Cervus elaphus hippelaphus*) became available online ^1^. The present paper presents the first *de novo* genomic draft of the tule elk. This California-endemic elk subspecies underwent a major genetic bottleneck when its numbers were reduced to as few as three individuals in the 1870s ^2, 3^. Although their numbers have increased to >5,000 today ^4^, the historical bottleneck nevertheless left its mark on the elk’s genome, rendering it more homozygous than other elk subspecies.

Our motivation for generating a genomic resource for the tule elk was to create a reference for identifying single nucleotide polymorphisms (SNPs) to develop assays to monitor elk population abundance and for related population genetic applications. Due to the relatively low coverage generated in this work (40X overall with an average of 10X coverage from each individual), we used the MEGAHIT metagenome assembler, which has been found to perform well on low-quality or low-coverage DNA sequencing in bacteria ^5^.

## Methods

### Sample collection and library prep

Elk were selected from four geographically distinct populations across northern California to maximize genomic diversity (San Luis Reservoir, California Valley, American Canyon, and the San Luis National Wildlife Refuge ^4^). Genomic DNA was extracted from skin biopsies, which were obtained by the California Department of Fish and Wildlife as part of their elk management activities ^4^. We extracted DNA from skin using Qiagen DNeasy blood & tissue kits (QIAGEN Inc., Valencia, CA), according to the manufacturer’s instructions. The DNA was then fragmented via sonication using a Bioruptor (Diagenode, Denville, NJ) to 300 to 400 base pairs (bp) prior to adapter ligation. After verification of fragment size range using agarose gel electrophoresis, NEBNext® Ultra™ DNA Library Prep Kit for Illumina® (New England Biolabs, Inc., Ipswich, MA) was used to ligate Illumina adapters. Multiplexed libraries were prepared using NEBNext Multiplex Oligos for Illumina (New England Biolabs) to individually barcode each of four individual elk. Barcodes were annealed using low-cycle polymerase chain reactions during library preparation. To assess library quality, trace analysis was performed using a Bioanalyzer 2100 (Agilent, Santa Clara, CA) and fluorometric DNA quantitation of libraries was performed using a Qubit fluorometer (Invitrogen, Carlsbad, CA) prior to equilibrating sample concentrations and pooling for sequencing. After library quality control, the four samples were pooled in equimolar concentrations and submitted for paired-end sequencing. Samples were sequenced on an Illumina HiSeq 3000 at the DNA Technologies and Expression Analysis Core of the UC Davis Genome Center.

### Bioinformatics processing

Sequencing quality on demultiplexed reads was evaluated using FastQC v0.11.3 (RRID:SCR_014583) ^6^. The Illumina TruSeq3-PE sequencing adapters were removed using Trimmomatic v0.30 (RRID:SCR_011848) ^7^ with the ILLUMINACLIP parameter set to TruSeq3-PE.fa:2:40:15. The TruSeq3-PE.fa sequence was downloaded from https://anonscm.debian.org/cgit/debian-med/trimmomatic.git/plain/adapters/TruSeq3-PE.fa. LEADING and TRAILING parameters were set to 2, resulting in the removal of bases with a quality score of 2 or less according to a phred33 quality scoring matrix. The SLIDINGWINDOW parameter of 4:2 was used to clip reads once the quality score fell below 2 within the window. The MINLENGTH parameter set to 25 dropped any reads that fell below that length due to quality trimming. The demultiplexed, quality-filtered reads were interleaved using the interleave-reads.py script in khmer v2.0 (RRID:SCR_001156) ^8^. The assembly was performed using MEGAHIT v1.0.5 ^9^ on interleaved quality filtered reads. Genome statistical analysis was done using QUAST v3.0 (RRID:SCR_001228) ^10^. All code used is publicly available at https://github.com/dib-lab/2017-tule-elk/.

## Results

We obtained 377,980,276 demultiplexed 150 bp paired-end raw reads, containing a total of 113.394 Gbp of sequence, from which 99.830 Gbp (88%) had quality scores ≥ Q30 (average quality score = 37.2), or approximately 40X coverage of the approximately 3 Gbp tule elk genome. Sequence assembly resulted in the generation of a total genome sequence size of 2.395 Gbp. Reads were assembled into 602,862 contiguous sequences ("contigs") averaging 3,973 bp in length with a minimum contig length of 201 bp. The G+C content of the genome was 41.55%. The N50 was 6,885 bp and maximum contig length was 72,391 bp. Additional assembly statistics are available in Table 1. No contigs (e.g. under a certain size or likely to reflect repeats) were removed from the assembly.

**Table 1.** Quality metrics on tule elk ( *Cervus canadensis nannodes*) assembly, as generated with QUAST v3.0.

Metric	Tule elk assembly
# contigs (≥ 200 bp)	1,367,218
# contigs ≥ 500 bp	602,862
# contigs (≥ 1000 bp)	460,702
# contigs (≥ 5000 bp)	160,229
# contigs (≥ 10000 bp)	51,790
# contigs (≥ 25000 bp)	2,606
# contigs (≥ 50000 bp)	36
Total length (≥ 200 bp)	2,607,088,486
Total length (≥ 1000 bp)	2,295,163,580
Total length (≥ 5000 bp)	1,531,314,985
Total length (≥ 10000 bp)	771,863,493
Total length (≥ 25000 bp)	80,157,993
Total length (≥ 50000 bp)	2,056,962
Largest contig	72,391
Total length	2,395,105,945
GC	41.55%
N50	6,885
N75	3,646
L50	103,346
L75	222,107
# N's per 100 kbp	0

This genome can serve as the basis for further genomic work on tule elk and other cervids, such as the development of a SNP assay to track elk population movement across increasingly developed northern Californian terrain. Furthermore, it is one of the first whole genome assemblies available from the family Cervidae, providing a useful interim reference genome for bioinformatic analyses on other deer and elk species.

## Data availability

The data referenced by this article are under copyright with the following copyright statement: Copyright: © 2017 Mizzi JE et al.

Data associated with the article are available under the terms of the Creative Commons Zero "No rights reserved" data waiver (CC0 1.0 Public domain dedication). 

Raw reads are available in the SRA under the BioProject ID PRJNA345218. The genome draft is available at https://doi.org/10.6084/m9.figshare.5382565.v1 ^11^.

Code used in this study have been archived at http://doi.org/10.5281/zenodo.887935 ^12^

## References

[1] https://www.ncbi.nlm.nih.gov/genome/10790, accessed 11/29/17.

[2] McCullough DR: The Tule Elk: Its History, Behavior, and Ecology. elibrary.ru.1969 Reference Source

[3] Sacks BN Lounsberry ZT Kalani T: Development and Characterization of 15 Polymorphic Dinucleotide Microsatellite Markers for Tule Elk Using HiSeq3000. *J Hered.* 2016;107(7):666–669. 10.1093/jhered/esw069 27638816

[4] Hobbs JH: Draft Conservation and Management Plan for Elk. Report to the California Department of Fish and Wildlife.2014;41.

[5] Seitz A Nieselt K: Improving ancient DNA genome assembly. *PeerJ.* 2017;5:e3126. 10.7717/peerj.3126 28392981 PMC5384568

[6] Andrews S: FastQC: a quality control tool for high throughput sequence data.2010 Reference Source

[7] Bolger AM Lohse M Usadel B: Trimmomatic: a flexible trimmer for Illumina sequence data. *Bioinformatics.* 2014;30(15):2114–20. 10.1093/bioinformatics/btu170 24695404 PMC4103590

[8] Crusoe MR Alameldin HF Awad S: The khmer software package: enabling efficient nucleotide sequence analysis [version 1; referees: 2 approved, 1 approved with reservations]. *F1000Res.* 2015;4:900. 10.12688/f1000research.6924.1 26535114 PMC4608353

[9] Li D Luo R Liu CM: MEGAHIT v1.0: A fast and scalable metagenome assembler driven by advanced methodologies and community practices. *Methods.* 2016;102:3–11. 10.1016/j.ymeth.2016.02.020 27012178

[10] Gurevich A Saveliev V Vyahhi N: QUAST: quality assessment tool for genome assemblies. *Bioinformatics.* 2013;29(8):1072–75. 10.1093/bioinformatics/btt086 23422339 PMC3624806

[11] Mizzi J Lounsberry ZT Brown CT: Tule Elk Draft Genome. *figshare.* 2017 Data Source 10.12688/f1000research.12636.1 PMC5747339 29333237

[12] Mizzi J: dib-lab/2017-tule-elk: Ready for publication. *Zenodo.* 2017 Data Source

