# Supplementary material for: Draft genome of tule elk Cervus canadensis nannodes
Source: F1000Res. 2017 Dec 11;6:1691. Originally published 2017 Sep 15. [Version 2] doi: 10.12688/f1000research.12636.2 (PMC5747339; doi:10.12688/f1000research.12636.2)
Supplement: Peer review history

# Peer review: Referee response for version 2

**DOI:** 10.5256/f1000research.14577.r28939

**Published:** 2017-12-18

**Authors:** Rudiger Brauning

**Affiliations:** 1 Invermay Agricultural Centre, AgResearch, Mosgiel, New Zealand

I'm happy with the changes made, no further comments.

I have read this submission. I believe that I have an appropriate level of expertise to confirm that it is of an acceptable scientific standard.

# Peer review: Referee response for version 1

**DOI:** 10.5256/f1000research.13682.r27606

**Published:** 2017-11-16

**Authors:** Rudiger Brauning

**Affiliations:** 1 Invermay Agricultural Centre, AgResearch, Mosgiel, New Zealand

The authors describe the generation of a draft assembly for tule elk in the style of a brief genome announcement. For SNP detection and primer design this assembly is fine. It could e.g. be used in combination with Genotyping by Sequencing on additional individuals.

 Materials and methods are sound and provided in full.

 However a quick search of NCBI's taxonomy resource reveals that since June 2017 there is a genome assembly for red deer available https://www.ncbi.nlm.nih.gov/genome/10790. The authors therefore cannot claim to present the first whole genome assembly from the family Cervidae. Please change that statement.

**Suggested further improvements:**

**Results**

 I would have liked to see a figure for the total amount of sequence after filtering as a simple way of showing how good or bad the sequence run was.

 Table 1's readability would be improved by getting all figures to align right.

 I'd also recommend to add another assembly metric to look at the gene content; either using something like BUSCO or by mapping the refseq sequences of a related, well annotated species (e.g. cattle) against the draft genome.

**Methods**

****

**Sample collection and library prep**

 I see that each individual has two tissue samples. The authors entered a sample ID into the 'tissue' field of NCBI's BioSample database. I'd recommend removing this and adding the animal ID in the 'isolate' field.

 Please expand the entries in the 'isolation source' field. It says e.g. "Am. Cyn" which probably means American Canyon.

**Bioinformatics processing**

 Checking the code I believe the statement "LEADING, TRAILING, and SLIDING parameters were set to 2" should read "LEADING and TRAILING parameters were set to 2".

I have read this submission. I believe that I have an appropriate level of expertise to confirm that it is of an acceptable scientific standard, however I have significant reservations, as outlined above.

# Response

**Published:** 2017-12-05

**Authors:** Jessica Mizzi

**Affiliations:** UC Davis, USA

Thank you for your review of this paper. Version 2 has been edited to reflect the presence of the red deer genome and a citation to that genome has been made. Table 1 has been reformatted for readability. The changes you’ve requested to the NCBI BioSample entry have been made. The trimmomatic code in the Bioinformatics Processing section has been edited to remove the erroneous “SLIDING” parameter. We’ve added text to the first sentence of the results section that describes the quality of sequence data in terms of standard quality scores. We opted not to provide details on the gene content relative to a related genome as we felt this could be done more comprehensively in the future once the red deer genome has been published and peer-reviewed.

# Peer review: Referee response for version 1

**DOI:** 10.5256/f1000research.13682.r26607

**Published:** 2017-10-25

**Authors:** Steve Olsen

**Affiliations:** 1 Infectious Bacterial Diseases Research Unit, National Animal Disease Center, ARS-USDA, Ames, IA, USA

This article describes the generation of a draft genome (40X coverage from 4 animals) of the tule elk (Cervus elaphus nannodes). The research methods are fairly standard for the Illumina sequencing used.  At 602,862 contigs, the genome is very prelminary and will require quite a bit of additional work in order for it to be applicable to a wide range of applications.  The report basically falls into a category of a genome announcement.

I have read this submission. I believe that I have an appropriate level of expertise to confirm that it is of an acceptable scientific standard.

# Response

**Published:** 2017-12-05

**Authors:** Jessica Mizzi

**Affiliations:** UC Davis, USA

Thank you for your review of this paper.
